# Bambusicolous *Arthrinium* Species in Guangdong Province, China

**DOI:** 10.3389/fmicb.2020.602773

**Published:** 2020-12-14

**Authors:** Indunil C. Senanayake, Jayarama D. Bhat, Ratchadawan Cheewangkoon, Ning Xie

**Affiliations:** ^1^Guangdong Provincial Key Laboratory for Plant Epigenetics, College of Life Science and Oceanography, Shenzhen University, Shenzhen, China; ^2^Shenzhen Key Laboratory of Laser Engineering, College of Optoelectronic Engineering, Shenzhen University, Shenzhen, China; ^3^Formerly, Department of Botany, Goa University, Taleigão, India; ^4^No. 128/1J, Azad Co-Op Housing Society, Curca, India; ^5^Department of Entomology and Plant Pathology, Faculty of Agriculture, Chiang Mai University, Chiang Mai, Thailand

**Keywords:** Apiosporaceae, bamboo, fungal taxonomy, new locality records, novel species

## Abstract

A survey of bambusicolous fungi in Bijiashan Mountain Park, Shenzhen, Guangdong Province, China, revealed several *Arthrinium*-like taxa from dead sheaths, twigs, and clumps of *Bambusa* species. Phylogenetic relationships were investigated based on morphology and combined analyses of the internal transcribed spacer region (ITS), large subunit nuclear ribosomal DNA (LSU), beta tubulin (β-tubulin), and translation elongation factor 1-alpha (tef 1-α) gene sequences. Based on morphological characteristics and phylogenetic data, *Arthrinium acutiapicum* sp. nov. and *Arthrinium pseudorasikravindrae* sp. nov. are introduced herein with descriptions and illustrations. Additionally, two new locality records of *Arthrinium bambusae* and *Arthrinium guizhouense* are described and illustrated.

## Introduction

*Arthrinium* Kunze is accommodated in Apiosporaceae, Xylariales, which is morphologically different from other xylariaceous genera by the presence of basauxic conidiophores and dark, aseptate, globose to lenticular conidia with a hyaline rim or germ slit (Minter, 1985; Petrini and Müller, 1986; Singh et al., 2012; Jiang et al., 2018; Pintos et al., 2019). Basauxic conidiophores simply mean elongation of conidiogenous cells from the basal growing point after formation of a single, terminal blastic conidium at its apex (Cole, 1986).

*Arthrinium* species are distributed worldwide in various hosts as endophytes, epiphytes, or saprobes, as well as plant pathogens on some commercial crops and ornamentals (Agut and Calvo, 2004; Ramos et al., 2010; Crous and Groenewald, 2013; Sharma et al., 2014; Senanayake et al., 2015; Wijayawardene et al., 2020). Also, species of *Arthrinium* (Schmidt and Kunze, 1817) inhabit a wide range of substrates, i.e., air, soil debris, lichens, marine algae (Agut and Calvo, 2004; Senanayake et al., 2015; Dai et al., 2016; Luo et al., 2019), and even human tissues (Sharma et al., 2014). The genus *Arthrinium* morphologically differs from other xylariaceous anamorphic genera by the presence of basauxic conidiogenous cells which arise from conidiophore mother cells (Schmidt and Kunze, 1817; Minter, 1985).

The commonly reported diseases by *Arthrinium* species are kernel blight of barley and brown culm streak of *Phyllostachys praecox* by *A. arundinis*, damping-off of wheat by *A. sacchari*, and culm rot of bamboos and *Phyllostachys viridis* by *A. phaeospermum* (Martínez-Cano et al., 1992; Mavragani et al., 2007; Chen et al., 2014; Li et al., 2016). Additionally, the causative agent of cutaneous infections of humans has been reported as *A. phaeospermum* (Rai, 1989; Zhao et al., 1990; De Hoog et al., 2000; Crous and Groenewald, 2013). Some *Arthrinium* species produce bioactive compounds with pharmacological and medicinal properties (Hong et al., 2015), while some produce industrially important enzymes (Shrestha et al., 2015). Crous and Groenewald (2013) reviewed the genus *Arthrinium* based on morphology and multigene phylogeny. There are several subsequent studies providing additions to the genus (Singh et al., 2012; Sharma et al., 2014; Crous et al., 2015; Senanayake et al., 2015; Dai et al., 2016, 2017; Hyde et al., 2016; Réblová et al., 2016; Jiang et al., 2018, 2020; Wang et al., 2018; Pintos et al., 2019).

Several studies revealed bambusicolous *Arthrinium* species from Poaceae and Cyperaceae host plants in China (Dai et al., 2016, 2017; Jiang et al., 2018, 2020; Wang et al., 2018), and several *Arthrinium*-like taxa on dead leaves, clumps, and twigs of bamboo were collected from Shenzhen (China) during this study. The aims of this study are identifying these *Arthrinium*-like taxa based on morphology and phylogeny and describe and illustrate them. Phylogenetic relationships were investigated based on DNA sequence data of the internal transcribed spacer region (ITS), large subunit nuclear ribosomal DNA (LSU), beta tubulin (β-tubulin), and translation elongation factor 1-alpha (tef 1-α), and two new *Arthrinium* species from bamboo are introduced as *Arthrinium pseudorasikravindrae* and *A. acutiapicum* and two locality records, *Arthrinium bambusae* and *Arthrinium guizhouense*, are described and illustrated.

## Materials and Methods

### Sample Collection and Fungal Isolation

Fresh specimens of *Arthrinium*-like taxa were collected from Bijiashan Mountain Park, Shenzhen, Guangdong Province, China, in September–October 2018, and the collection site has a tropical climate with abundant sunshine and rainfall all year round. The yearly average temperature is 22°C and vegetative type is tropical evergreen monsoon forests (Li et al., 2015). Specimens were brought to the laboratory in paper bags and they were examined under a stereomicroscope (Carl Zeiss Discovery V8), and blackish conidial mass and fruit bodies were observed. The fruit bodies were studied and photographed using a stereomicroscope fitted with a camera (ZEISS Axiocam 208). The micromorphological characters were studied and photographed using a compound microscope (Nikon Eclipse 80i) fitted with a digital camera (Canon 450D). All microscopic measurements such as the length of the conidiophores, conidiogenous cells, and conidia were made with Tarosoft image framework (v. 0.9.0.7).

Single conidial isolation was carried out following the method described by Senanayake et al. (2020a). Germinated conidia were aseptically transferred into fresh potato dextrose agar (PDA) plates, incubated at 20°C to obtain pure cultures, and later transferred to PDA slants and stored at 4°C for further study. Colony characteristics were recorded from cultures grown on PDA. Additionally, pure cultures were inoculated in 2% PDA together with sterilized bamboo leaves and incubated at 25°C for sporulation.

Fungarium materials are deposited in the Herbarium of Cryptogams, Kunming Institute of Botany, Academia Sinica (HKAS), and all the ex-type living cultures are deposited at the Culture Collection of Kunming Institute of Botany (KUMCC). Index Fungorum numbers^1^ were obtained for the new strains.

### DNA Extraction, PCR Amplification, and Sequencing

Fresh fungal mycelium grown on PDA for 2 weeks at 20°C in the dark was used for DNA extraction using fungal genomic DNA extraction kit (Biospin DNA Extraction Kit, Bioer Technology, Co. Ltd., Hangzhou, China) following the manufacturer’s protocols. Polymerase chain reactions (PCR) and sequencing were carried out for the following loci: the complete ITS region with the primer pair of ITS1/ITS4 (White et al., 1990); the LSU ribosomal DNA, amplified and sequenced as a single fragment with primers LR0R/LR5 (Vilgalys and Hester, 1990); the tef 1-α gene with primers EF1-728F/EF2 (Carbone and Kohn, 1999; Rehner, 2001); and the β-tubulin gene with primers bt2a and bt2b (Glass and Donaldson, 1995).

The PCR amplification reactions were carried out with the following protocol. The total volume of the PCR reaction was 25 μl reaction volume containing 1 μl of DNA template, 1 μl of each forward and reverse primer, 12.5 μl of 2 × PCR Master Mix, and 9.5 μl of double-distilled sterilized water (ddH_2_O). The reaction was conducted by running for 35 cycles following the condition of Senanayake et al. (2020b). The PCR products were observed on 1% agarose electrophoresis gel stained with ethidium bromide. Purification and sequencing of PCR products were carried out at Sunbiotech Company, Beijing, China. Sequence quality was checked and sequences were condensed with DNASTAR Lasergene v.7.1. Sequences derived in this study were deposited in GenBank and accession numbers were obtained (Table 1).

**TABLE 1:** Details of the isolates used in the phylogenetic analyses.

**Species**	**Strains**	**Substrate**	**Location**	**GenBank Accession Number**			
				**ITS**	**LSU**	**β-Tubulin**	**tef 1-α**
***Arthrinium acutiapicum***	**KUMCC 20-0209**	***Bambusa bambos***	**China**	**MT946342**	**MT946338**	**MT947365**	**MT947359**
***A. acutiapicum***	**KUMCC 20-0210**	***Bambusa bambos***	**China**	**MT946343**	**MT946339**	**MT947366**	**MT947360**
*A. aquaticum*	MFLU 18-1628	Submerged wood	China	MK828608	MK835806	N/A	N/A
*A. arundinis*	CBS 450.92	N/A	N/A	AB220259	N/A	AB220306	N/A
*A. arundinis*	CBS 114316	*Hordeum vulgare*	Iran	KF144884	KF144928	KF144974	KF145016
*A. arundinis*	AP11118A	*Bambusa* sp.	Spain	MK014868	MK014835	MK017974	MK017945
*A. aureum*	CBS 244.83	*Phragmites australis*		AB220251	KF144935	KF144981	KF145023
*A. balearicum*	CBS 145129	Poaceae sp.	Spain	MK014869	MK014836	MK017975	MK017946
*A. bambusae*	CGMCC 3.18335	Bamboo	China	KY494718	KY494794	KY705186	KY806204
***A. bambusae***	**KUMCC 20-0207**	***Bambusa dolichoclada***	**China**	**MT946346**	**MT946340**	**MT947370**	**MT947363**
*A. bambusae*	LC7107	Bamboo	China	KY494719	KY494795	KY705187	KY705117
*A. camelliae-sinensis*	CGMCC 3.18333	*Camellia sinensis*	China	KY494704	KY494780	KY705173	KY705103
*A. camelliae-sinensis*	LC8181	*Brassica campestris*	China	KY494761	KY494837	KY705229	KY705157
*A. caricicola*	CBS 145127	*Carex ericetorum*	Germany	MK014871	MK014838	MK017977	MK017948
*A. chinense*	CFCC 53036	*Fargesia qinlingensis*	China	MK819291	N/A	MK818547	MK818545
*A. chinense*	CFCC 53037	*Fargesia qinlingensis*	China	MK819292	N/A	MK818548	MK818546
*A. curvatum*	CBS 145131	*Carex* sp.	Germany	MK014872	MK014839	MK017978	MK017949
*A. descalsii*	CBS 145130	*Ampelodesmos mauritanicus*	Spain	MK014870	MK014837	MK017976	MK017947
*A. dichotomanthi*	CGMCC 3.18332	*Dichotomanthus tristaniaecarpa*	China	KY494697	KY494832	KY705167	KY705096
*A. dichotomanthi*	LC8175	*Dichotomanthus tristaniaecarpa*	China	KY494755	KY494831	KY705223	KY705151
*A. esporlense*	CBS 145136	*Phyllostachys aurea*	Spain	MK014878	MK014845	MK017983	MK017954
*A. euphorbiae*	IMI 285638b	*Bambusa* sp.	Bangladesh	AB220241	N/A	AB220288	NA
*A. gaoyouense*	CFCC 52301	*Phragmites australis*	China	MH197124	N/A	MH236789	MH236793
*A. gaoyouense*	CFCC 52302	*Phragmites australis*	China	MH197125	N/A	MH236790	MH236794
*A. garethjonesii*	JHB004	*Bambusa* sp.	China	KY356086	KY356091	N/A	N/A
*A. garethjonesii*	HKAS 96289	*Bambusa* sp.	China	NR_154736	NG_057131	N/A	N/A
*A. guizhouense*	LC5318	Air	China	KY494708	KY494784	KY705177	KY705107
***A. guizhouense***	**KUMCC 20-0206**	***Bambusa multiplex***	**China**	**MT946347**	**MT946341**	**MT947369**	**MT947364**
*A. guizhouense*	CGMCC 3.18334	Air	China	KY494709	KY494785	KY705178	KY705108
*A. gutiae*	CBS 135835	Gut of a grasshopper	India	KR011352	MH877577	KR011350	KR011351
*A. hispanicum*	IMI 326877	Maritime sand	Spain	AB220242	AB220336	AB220289	NA
*A. hydei*	KUMCC 16-0204	*Bambusa tuldoides*	China	KY356087	KY356092	N/A	NA
*A. hydei*	CBS 114990	Bamboo	China	KF144890	KF144936	KF144982	KF145024
*A. hyphopodii*	MFLUCC 15-0003	*Bambusa tuldoides*	China	KR069110	N/A	N/A	NA
*A. hyphopodii*	KUMCC 16-0201	Bamboo	China	KY356088	N/A	N/A	NA
*A. hysterinum*	ICPM6889	Bamboo	New Zealand	MK014874	MK014841	MK017980	MK017951
*A. hysterinum*	AP2410173	*Phyllostachys aurea*	Spain	MK014876	MK014843	N/A	N/A
*A. ibericum*	CBS 145137	*Arundo donax*	Portugal	MK014879	MK014846	MK017984	MK017955
*A. italicum*	CBS 145138	*Phragmites australis*	Spain	MK014880	MK014847	MK017985	MK017956
*A. italicum*	AP221017	*Phragmites australis*	Spain	MK014881	MK014848	MK017986	MK017957
*A. japonicum*	IFO 30500	*Carex despalata*	Japan	AB220262	AB220356	AB220309	N/A
*A. japonicum*	IFO 31098	*Carex despalata*	Japan	AB220264	AB220358	AB220311	N/A
*A. jatrophae*	MMI 00051	*Jatropha podagrica*	India	JQ246355	N/A	N/A	N/A
*A. jiangxiense*	CGMCC 3.18381	*Maesa* sp.	China	KY494693	N/A	KY705163	KY705092
*A. jiangxiense*	LC4578	*Camellia sinensis*	China	KY494694	KY494770	KY705164	KY705093
*A. kogelbergense*	CBS 113332	*Cannomois virgate*	South Africa	KF144891	KF144937	KF144983	KF145025
*A. kogelbergense*	CBS 113333	Restionaceae sp.	South Africa	KF144892	KF144938	KF144984	KF145026
*A. locutum-pollinis*	LC11683	*Brassica campestris*	China	MF939595	N/A	MF939622	MF939616
*A. longistromum*	MFLUCC 11-0479	Bamboo	Thailand	KU940142	KU863130	N/A	NA
*A. longistromum*	MFLUCC 11-0481	Bamboo	Thailand	KU940141	KU863129	N/A	NA
*A. longistromum*	MFLU 15-1184	*Bambusa* sp.	Thailand	NR_154716	N/A	N/A	NA
*A. malaysianum*	CBS 251.29	*Cinnamomum camphora*	N/A	KF144897	KF144943	KF144989	KF145031
*A. malaysianum*	CBS 102053	*Macaranga hullettii*	Malaysia	KF144896	KF144942	KF144988	KF145030
*A. marii*	CBS 497.90	Air	Spain	AB220252	KF144947	KF144993	KF145035
*A. marii*	CBS 114803	*Arundinaria hindsii*	China	KF144899	KF144945	KF144991	KF145033
*A. mediterranei*	IMI 326875	Air	Spain	AB220243	N/A	AB220290	NA
*A. neosubglobosa*	JHB006	Bamboo	China	KY356089	KY356094	N/A	NA
*A. neosubglobosa*	JHB007	Bamboo	China	KY356090	KY356095	N/A	NA
*A. obovatum*	CGMCC 3.18331	*Lithocarpus* sp.	China	KY494696	KY494834	KY705166	KY705095
*A. obovatum*	LC8177	*Lithocarpus* sp.	China	KY494757	KY494833	KY705225	KY705153
*A. ovatum*	CBS 115042	*Arundinaria hindsii*	China	KF144903	KF144950	KF144995	KF145037
*A. paraphaeospermum*	MFLUCC 13-0644	Bamboo	Thailand	KX822128	KX822124	N/A	NA
*A. phaeospermum*	CBS 114314	*Hordeum vulgare*	Iran	KF144904	KF144951	KF144996	KF145038
*A. phaeospermum*	CBS 114315	*Hordeum vulgare*	Iran	KF144905	KF144952	KF144997	KF145039
*A. phragmitis*	CPC 18900	*Phragmites australis*	Italy	KF144909	N/A	KF145001	KF145043
*A. phragmitis*	AP3218	*Phragmites australis*	Spain	MK014891	MK014858	MK017996	MK017967
*A. phragmitis*	AP2410172A	*Phragmites australis*	Spain	MK014890	MK014857	MK017995	MK017966
*A. piptatheri*	CBS 145149	*Piptatherum miliaceum*	Spain	MK014893	MK014860	N/A	MK017969
*A. pseudoparenchymaticum*	CGMCC 3.18336	Bamboo	China	KY494743	KY494830	KY705211	KY705139
*A. pseudoparenchymaticum*	LC8173	Bamboo	China	KY494753	KY494829	KY705221	KY705149
***A. pseudorasikravindrae***	**KUMCC 20-0208**	***Bambusa dolichoclada***	**China**	**MT946344**	**N/A**	**MT947367**	**MT947361**
***A. pseudorasikravindrae***	**KUMCC 20-0211**	***Bambusa dolichoclada***	**China**	**MT946345**	**N/A**	**MT947368**	**MT947362**
*A. pseudosinense*	CBS 135459	Bamboo	Netherlands	KF144910	KF144957	N/A	KF145044
*A. pseudospegazzinii*	CBS 102052	*Macaranga hullettii*	Malaysia	KF144911	KF144958	KF145002	KF145045
*A. pterospermum*	CBS 123185	*Machaerina sinclairii*	New Zealand	KF144912	KF144959	KF145003	NA
*A. pterospermum*	CBS 134000	*Machaerina sinclairii*	Australia	KF144913	KF144960	KF145004	KF145046
*A. puccinioides*	CBS 549.86	*Lepidosperma gladiatum*	Germany	AB220253	AB220347	AB220300	NA
*A. qinlingense*	CFCC 52303	*Fargesia qinlingensis*	China	MH197120	N/A	MH236791	MH236795
*A. qinlingense*	CFCC 52304	*Fargesia qinlingensis*	China	MH197121	N/A	MH236792	MH236796
*A. rasikravindrae*	NFCCI 2144	*Cissus* sp.	Netherlands	KF144914	N/A	N/A	NA
*A. rasikravindrae*	MFLUCC 11-0616	Bamboo	Thailand	KU940144	KU863132	N/A	NA
*A. sacchari*	CBS 212.30	*Phragmites australis*	UK	KF144916	KF144962	KF145005	KF145047
*A. sacchari*	CBS 301.49	Bamboo	Indonesia	KF144917	KF144963	KF145006	KF145048
*A. saccharicola*	CBS 191.73	Air	Netherlands	KF144920	KF144966	KF145009	KF145051
*A. saccharicola*	CBS 463.83	*Phragmites australis*	Netherlands	KF144921	KF144968	KF145010	KF145052
*A. serenense*	IMI 326869	N/A	Spain	AB220250	N/A	AB220297	NA
*A. serenense*	ATCC 76309	N/A	N/A	AB220240	N/A	AB220287	NA
*A. sporophleum*	CBS 145154	*Juncus* sp.	Spain	MK014898	MK014865	MK018001	MK017973
*A. subglobosum*	MFLUCC 11-0397	Bamboo	Thailand	KR069112	KR069113	N/A	NA
*A. subroseum*	LC7291	Bamboo	China	KY494751	KY494827	KY705219	KY705147
*A. subroseum*	CGMCC3.18337	Bamboo	China	KY494752	KY494828	KY705220	KY705148
*A. thailandicum*	MFLUCC 15-0199	Bamboo	Thailand	KU940146	KU863134	N/A	NA
*A. thailandicum*	MFLUCC 15-0202	Bamboo	Thailand	KU940145	KU863134	N/A	NA
*A. tintinnabula*	ICPM 6889	Bamboo	New Zealand	MK014874	MK014841	MK017980	MK017951
*A. trachycarpum*	CFCC 53038	*Trachycarpus fortune*	China	MK301098	N/A	MK303394	MK303396
*A. trachycarpum*	CFCC 53039	*Trachycarpus fortune*	China	MK301099	N/A	MK303395	MK303397
*A. vietnamense*	IMI 99670	*Citrus sinensis*	Vietnam	KX986096	KX986111	KY019466	NA
*A. xenocordella*	CBS 478.86	Soil	Zimbabwe	KF144925	KY494763	N/A	NA
*A. xenocordella*	CBS 595.66	Soil	Austria	KF144926	KF144971	KF145013	KF145055
*A. yunnanum*	MFLU 15-0002	*Phyllostachys nigra*	China	KU940147	KU863135	N/A	NA
*A. yunnanum*	CFCC 52312	Bamboo	China	MH191120	N/A	N/A	NA
*Pestalotiopsis hamaeropis*	CBS 237.38	N/A	Italy	MH855954	MH867450	KM199392	KM199474

*Newly obtained sequences are indicated in black bold. ATCC, The American Type Culture Collection, Virginia, United States; CBS, Westerdijk Fungal Biodiversity Institute, Utrecht, The Netherlands; CFCC, China Forestry Culture Collection, Beijing, China; CGMCC, China General Microbiological Culture Collection, Beijing, China; CPC, Culture collection of Pedro Crous, housed at the Westerdijk Fungal Biodiversity Institute; HKAS, Herbarium of Cryptogams, Kunming, China; ICPM, International Collection of Microorganisms from Plants, Auckland, New Zealand; IFO, Institute for Fermentation, Osaka; IMI, Culture collection of CABI Europe UK Centre, UK; KUMCC, Culture Collection of Kunming Institute of Botany, Kunming, China; MFLU, Mae Fah Luang University Herbarium, Chiang Rai, Thailand; LC, Working collection of Lei Cai, housed at CAS, China; MFLUCC, Mae Fah Luang University Culture Collection, Chiang Rai, Thailand; NFCCI, National Fungal Culture Collection of India.*

### Sequence Alignments and Phylogenetic Analyses

BLASTn searches were made using the newly generated sequences to assist in taxon sampling for phylogenetic analyses. Jiang et al. (2018, 2020), Wang et al. (2018), and Pintos et al. (2019) were followed to obtain sequences from GenBank that are listed in Table 1. The concatenated ITS, LSU, β-tubulin, and tef 1-α sequence dataset comprised 101 strains of *Arthrinium*, while the outgroup taxon was *Pestalotiopsis chamaeropis* (CBS 237.38). DNA sequences of the ITS, LSU, β-tubulin, and tef 1-α were aligned using the online version of MAFFT v. 7.036^2^ (Katoh et al., 2020) with default settings and manually adjusted using BioEdit 7.1.3 (Hall, 1999) to allow maximum alignment and minimum gaps. Further, single gene alignments were combined to obtain the final multiloci alignment that was containing 2,817 nucleotide characters, viz. 681 of ITS, 875 of LSU, 434 of β-tubulin, and 827 of tef 1-α. Both single and concatenated alignments were used for the analyses.

Maximum likelihood analyses were performed by RAxML (Stamatakis and Alachiotis, 2010) implemented in raxmlGUIv.1.5 (Silvestro and Michalak, 2012) using the ML + rapid bootstrap setting and the GTR + I + G model of nucleotide substitution with 1,000 replicates. The matrix was partitioned for the different gene regions included in the combined multilocus analyses.

For the Bayesian inference (BI) analyses, the optimal substitution model for the combined dataset was determined to be GTR + I + G using the MrModeltest software v. 2.2 (Nylander, 2004). The BI analyses was computed with MrBayes v. 3.2.6 (Ronquist et al., 2012) with four simultaneous Markov chain Monte Carlo chains from random trees over 10 M generations (trees were sampled every 500th generation).

The distribution of log-likelihood scores was observed to check whether sampling is in stationary phase or not, and Tracer v1.5 was used to check if further runs were required to reach convergence or not (Rambaut and Drummond, 2007). The Bayesian analyses lasted until the average standard deviation of split frequencies has a value less than 0.01, and the consensus tree and posterior probabilities were calculated after discarding the first 20% of the sampled trees as burn-in. The phylogram was visualized in FigTree v. 1.4 (Rambaut, 2009). All the phylogenetic trees derived from this study were deposited in TreeBase^3^ under accession number S27147.

## Results

### Phylogenetic Inferences

All individual trees generated under different criteria and from single gene datasets were essentially similar in topology and not significantly different from the tree generated from the concatenated dataset (not discussed herein). Additionally, this tree topology is similar to previous studies on *Arthrinium* (Dai et al., 2016; Jiang et al., 2018, 2020; Wang et al., 2018; Pintos et al., 2019).

**FIGURE 1:**
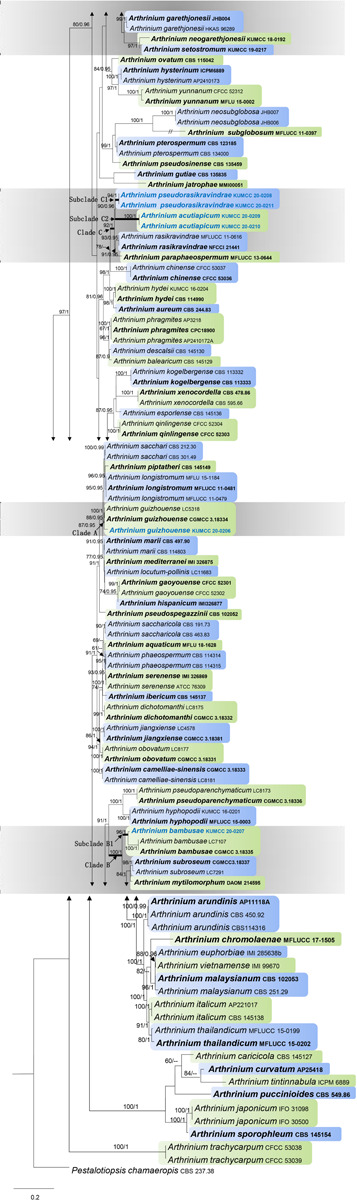
Phylogram generated from maximum likelihood analysis based on combined internal transcribed spacer region (ITS), large subunit nuclear ribosomal DNA (LSU), β-tubulin, and tef 1-α sequence data. Bootstrap support values greater than 50% and Bayesian posterior probabilities greater than 0.90 are given at the nodes. The tree is rooted with *Pestalotiopsis chamaeropis* (CBS 237.38). Ex-type strains are in bold and the newly obtained sequences are indicated in blue bold.

Maximum likelihood analysis of *Arthrinium* species in this study with 1,000 bootstrap replicates yielded the best ML tree (Figure 1) with the likelihood value of –29,933.362493 and the following model parameters: estimated base frequencies—*A* = 0.239654, *C* = 0.250345, *G* = 0.255054, and *T* = 0.254948; substitution rates—AC = 1.275584, AG = 2.530572, AT = 1.397969, CG = 1.184045, CT = 4.063803, and GT = 1.0; proportion of invariable sites—*I* = 0.203121; gamma distribution shape parameter—α = 0.54383. The alignment contained a total of 1,756 distinct alignment patterns and 28.72% of undetermined characters. After discarding the first 20% of generations, 36,000 trees remained from which 50% consensus trees and posterior probabilities (PP) were calculated (Figure 1). Maximum likelihood bootstrap values ≥ 60% and BI ≥ 0.95 are given at each node. Tree topologies of the ML and Bayesian analyses were similar to each other and there are no significant differences.

There are 101 *Arthrinium* strains in this study together with a new isolate that is introduced here. All the ex-type strains of *Arthrinium* species were included if available, and other authentic strains were selected when sequences from ex-type strains are unavailable. Our new isolate KUMCC 20-0206 clustered with the type strain of *A. guizhouense* (CGMCC 3.18334) and another representative strain (LC5318) with 87% ML and 0.95 PP support. This clade (clade A) has a close phylogenetic affinity to *Arthrinium longistromum*, *A*. *piptatheri*, and *A*. *sacchari* with 95% ML and 0.95 PP support. Two strains of *A*. *bambusae* (CGMCC 3.18335 and LC7107) and the new isolate KUMCC 20-0207 were grouped in a separate clade with 96% ML and 1.00 PP support. This clade (subclade B1) shares a monophyletic relationship to *Arthrinium garethgonesii*, *A*. *mytilomotphum*, *A*. *neogarethjonesii*, *A*. *setostromun*, and *A*. *subroseum* with strong bootstrap supports (100% ML, 1.00 PP, clade B, Figure 1). Two new isolates, KUMCC 20-0208 and KUMCC 20-0211, were monophyletic in subclade C1 (Figure 1) with 90% ML and 0.96 PP support. Subclade C2 is also monophyletic with two novel strains, viz. KUMCC 20–0209 and KUMCC 20–0210, which are sisters to subclade C1 with 90% ML and 0.96 PP support (clade C, Figure 1). With these four new strains, clade C shares a close phylogenetic affiliation to *A*. *paraphaeospermum* and *A*. *rasikravindrae*.

### Taxonomy

#### *Arthrinium acutiapicum* Senan. and Cheew. sp. nov. Figure 2

**FIGURE 2:**
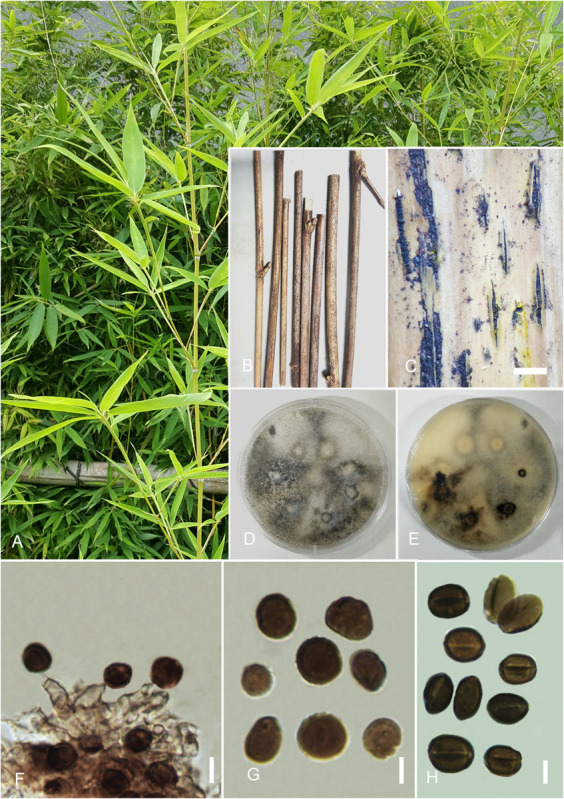
*Arthrinium acutiapicum* (HKAS 107673). **(A)** Host. **(B)** Fungarium specimen. **(C)** Conidiomata on substrate. **(D)** Surface view of culture on potato dextrose agar (PDA). **(E)** Reverse view of culture on PDA. **(F)** Conidia and conidiogenous cells. **(G,H)** Conidia. Scale bars: **(C)** = 500 μm, **(F–H)** = 10 μm.

Index Fungorum number: IF557868
Etymology: Species epithet “acuti” refers to pointed and “apicum” refers to apex of conidiogenous cells.
Holotype: HKAS 107673

*Saprobic* on dead twigs of *Bambusa bambos* (L.) Voss. *Hyphae* 1.5–2.5 μm diam., hyaline, branched, septate, sparse. *Sexual morph*: undetermined. *Asexual morph*: *Conidiomata* 350–450 μm, pycnidial, immersed, aggregated, scattered, subglobose, ostiolate, black, coriaceous. *Conidiophores* reduced to conidiogenous cells. *Conidiogenous cells* 4–7 × 2–3 μm ($\bar{x}$ = 6.3 × 2.1 μm, *n* = 30), holoblastic, develop from conidiophore mother cells, erect, basauxic, cylindrical to ampulliform, apex pointed and hyaline, smooth and thick-walled, pale brown. *Conidia* 7.5–10 × 8.5–12 μm ($\bar{x}$ = 9.3 × 11.9 μm, *n* = 30), globose in surface view, subglobose to oval in side view, apex and base blunted, smooth-walled, brown to dark brown, with a dark equatorial slit.

##### Culture Characteristics

Colonies grew on PDA at 20°C in the dark attenuated 2 cm diam., within 7 days, flat, circular, entire margin, wooly, with abundant aerial mycelia, white in surface view and off-white to yellow in reverse. Sporulation occurred after 10 days on PDA incubated at 20°C in the dark without any host substrate. Conidia seem black mass and well spread on culture.

##### Specimen Examined

China, Guangdong Province, Shenzhen City, Futian District, northwest of Futian, Bijiashan Park, on dead twigs of *B. bambos* (L.) Voss (Poaceae), 23 September 2018, IS, SI 86 (HKAS 107673, holotype), ex-type culture, KUMCC 20-0210; *ibid* 15 October 2018, IS, SI 86-1 (HKAS 107674, paratype), ex-paratype culture KUMCC 20-0211.

##### Notes

*Arthrinium acutiapicum* forms a distinct subclade (subclade C2, Figure 1) with strong bootstrap support values (ML/PP = 90/0.96) in our phylogenetic analysis, which is a sister to the newly introduced species *A. pseudorasikravindrae*. Additionally, *A. acutiapicum* shows close phylogenetic affinities to *A. paraphaeospermum*, *A. pseudorasikravindrae*, and *A. rasikravindrae* in clade C (Figure 1) and *A. chinense*. Blast results of ITS, LSU, β-tubulin, and tef 1-α sequences of *A. acutiapicum* show high similarity to *A. hydei*, *A. paraphaeospermum*, and *A. rasikravindrae.* Morphologically, *A. acutiapicum* is distinct from *A. pseudorasikravindrae* by the reduction of conidiophores to conidiogenous cells, cylindrical to ampulliform, pale brown conidiogenous cells with pointed, hyaline apex and brown to dark brown, smooth-walled conidia with dark equatorial slit. Additionally, *A. acutiapicum* is distinct from *A. rasikravindrae* by the reduction of conidiophores to pale brown conidiogenous cells and dimorphous, acropleurogenously arising conidia.

#### *Arthrinium bambusae* M. Wang and L. Cai, in Wang et al. (2018) Figure 3

**FIGURE 3:**
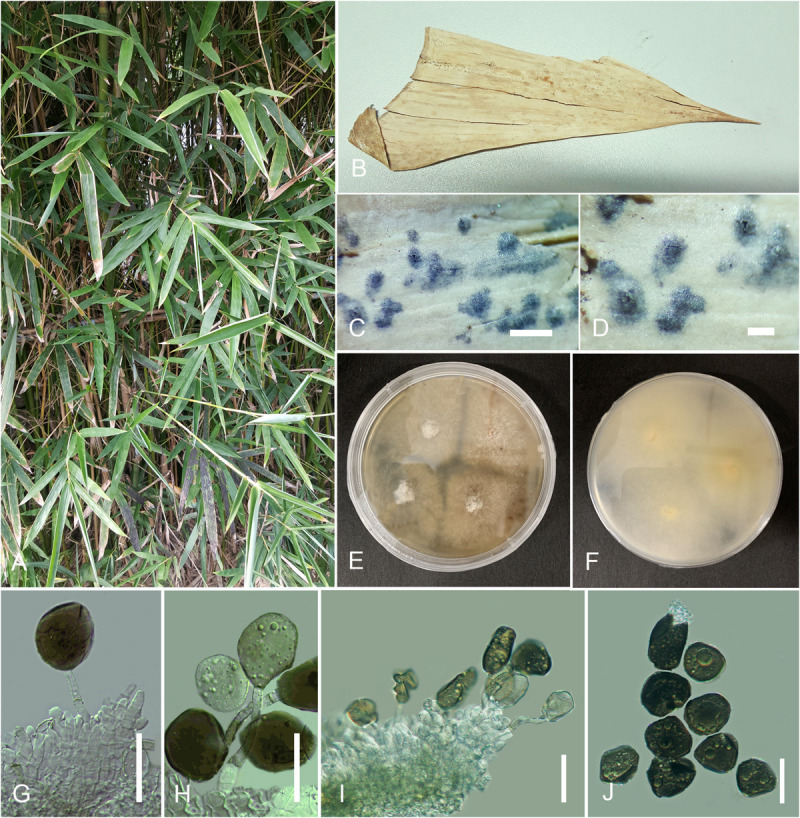
*Arthrinium bambusae* (HKAS 107671). **(A)** Host. **(B)** Fungarium specimen. **(C,D)** Conidiomata on substrate. **(E,F)** Culture on potato dextrose agar (PDA; surface and reverse view). **(G–I)** Conidia and conidiogenous cells. **(J)** Conidia. Scale bars: **(C,D)** = 100 μm, **(G–J)** = 15 μm.

Index Fungorum number: IF 824906

*Saprobic* on sheath of *Bambusa dolichoclada* Hayata. *Hyphae* 1–3 μm diam., hyaline, branched, septate, sparse. *Sexual morph*: undetermined. *Asexual morph*: appears as black, spotty patches on host surface. *Conidiomata* immersed, pycnidial, scattered, globose to slightly conical, ostiolate, black, coriaceous. *Conidiomatal wall* thin, comprising several layers of black, large cells of *textura angularis*. *Conidiophores* reduced to conidiogenous cells or rarely inconspicuous with cylindrical, thick-walled, hyaline. *Conidiogenous cells* 5–12 × 3–10 μm ($\bar{x}$ = 8.6 × 4 μm, *n* = 30), basauxic, holoblastic, develop from conidiophore mother cells, with periclinal thickening, doliiform to ampulliform or lageniform, erect, aggregated in clusters on hyphae, hyaline to pale brown, smooth. *Conidia* 10.5–17.5 × 8–15 μm ($\bar{x}$ = 15 × 12 μm, *n* = 30), subglobose to ellipsoid, guttulate, smooth to finely roughened, olivaceous to dark brown.

##### Culture Characteristics

Colonies grew on PDA at 20°C in the dark attenuated 2 cm diam., within 7 days, flat, spreading, margin circular, with abundant aerial mycelia, surface and reverse white to off-white.

##### Specimen Examined

China, Guangdong Province, Shenzhen City, Futian District, northwest of Futian, Bijiashan Park, on sheath of *B. dolichoclada* Hayata (Poaceae), 23 September 2018, IS, SI 80 (HKAS 107671), living culture, KUMCC 20-0207.

##### Notes

*Arthrinium bambusae* was introduced by Wang et al. (2018) from Guangdong Province, China, where our collection also was obtained. However, the exact locality is not mentioned in the original description there. The morphology of our collection was obtained from fungal structures on the host specimen, while Wang et al. (2018) had described the fungus from sporulated cultures. However, the morphology of our collection is similar to the holotype. Phylogenetically, *A. bambusae* clusters with *A. garethjonesii*, *A. neogarethjonesii*, *A. mytilomorphum*, *A. setostromum*, and *A. subroseum* with strong bootstrap value (ML/PP = 100/1), and the *A. bambusae* isolate (KUMCC 20-0207) clustered well with the ex-type culture (ML/PP = 96/1).

#### *Arthrinium guizhouense* M. Wang and L. Cai, in Wang et al. (2018)Figure 4

**FIGURE 4:**
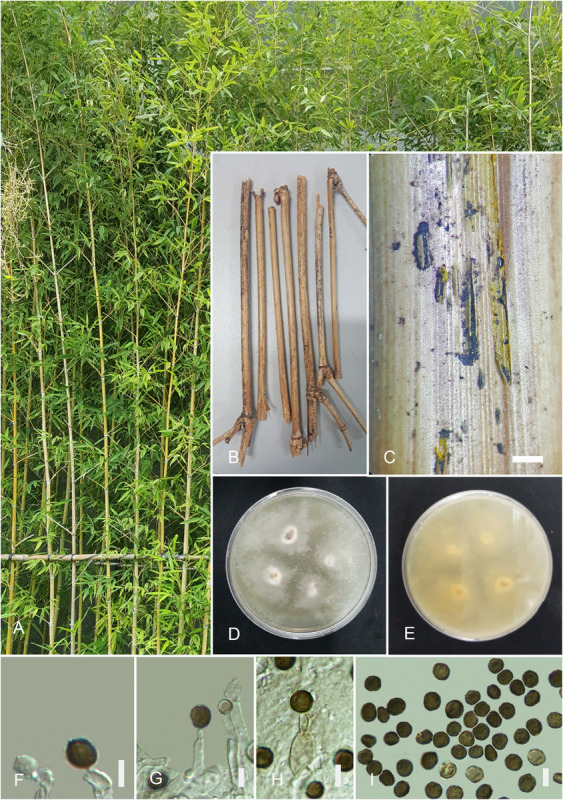
*Arthrinium guizhouense* (HKAS 107672). **(A)** Host. **(B)** Fungarium specimen. **(C)** Conidiomata on substrate. **(D)** Surface view of culture on potato dextrose agar (PDA). **(E)** Reverse view of culture on PDA. **(F–H)** Conidia and conidiogenous cells. **(I)** Conidia. Scale bars: **(C)** = 1,000 μm, **(F–I)** = 5 μm.

Index Fungorum number: IF824909

*Saprobic* on dead twigs of *Bambusa multiplex* (Lour.) Raeusch. ex Schult. f., appear as black mass coming out from ruptured bark. *Hyphae* hyaline, branched, septate, 1.5–6 μm diam. *Sexual morph*: undetermined. *Asexual morph*: *Conidiomata* 450–350 μm, picnidial, semi-immersed, aggregated, scattered, globose, ostiolate, black, coriaceous. *Conidiophores* reduced to conidiogenous cells. *Conidiogenous cells* 4–8 × 3–5 μm ($\bar{x}$ = 5.3 × 4 μm, *n* = 30), develop from conidiophore mother cells, erect, subglobose, basauxic, ampulliform or doliiform, hyaline to pale brown, smooth. *Conidia* 5–7 × 4–7 μm ($\bar{x}$ = 6.3 × 5.7 μm, *n* = 30), globose or subglobose, smooth to finely roughened, dark brown to black, with a longitudinal, hyaline, thin, germ slit.

##### Culture Characteristics

Colonies grew on PDA at 20°C in the dark attenuated 2 cm diam., within 5 days, flat, wooly, margin circular, with slight aerial mycelia, surface initially white, becoming grayish white and reverse yellowish white.

##### Specimen Examined

China, Guangdong Province, Shenzhen City, Futian District, northwest of Futian, Bijiashan Park, on twigs of *B. multiplex* (Lour.) Raeusch. ex Schult. f. (Poaceae), 23 September 2018, I.C. Senanayake, SI 84 (HKAS 107672), living culture, KUMCC 20-0206.

##### Notes

NCBI blast result for β-tubulin sequences of this isolate gives high sequence similarities to *A. guizhouense* (99.55%), *A. sacchari* (98.25%), *A. arundinis* (98.25%), and *A. marii* (95.62%) while *A. guizhouense* (93.41%) and *A. marii* (92.94%) for tef 1-α. Additionally, high blast similarities for ITS loci are *A. marii* (99.54%), *A. sacchari* (99.22%), *A. phaeospermum* (99.20%), *A. pseudospegazzinii* (98.13%), *A. longistromum* (98.72%), and *A. guizhouense* (99.83%) while *A. marii* (100%), *A. sacchari* (100%), *A. guizhouense* (100%), and *Apiospora montagnei* (100%) for LSU. In the phylogenetic analysis, this isolate (KUMCC 20-0206) clusters with the ex-holotype strain of *A. guizhouense* (CGMCC3.18334) with moderate support value (ML/PP = 87/0.95). Morphologically, this collection is closely similar to the holotype specimen of *A. guizhouense* having brown to black, smooth to finely roughened, globose or subglobose, conidia with pale brown, subglobose, ampulliform or doliiform conidiogenous cells. However, the holotype of *A. guizhouense* has been collected from the air in karst cave in Guizhou Province, China, and this collection was obtained from bamboo twigs in Guangdong Province. Hence, HKAS 107672 is identified as *A. guizhouense* based on morphology and phylogeny. This is the first record of *A. guizhouense* in Guangdong Province and on bamboo.

#### *Arthrinium pseudorasikravindrae* Senan., and Cheew. sp. nov. Figure 5

**FIGURE 5:**
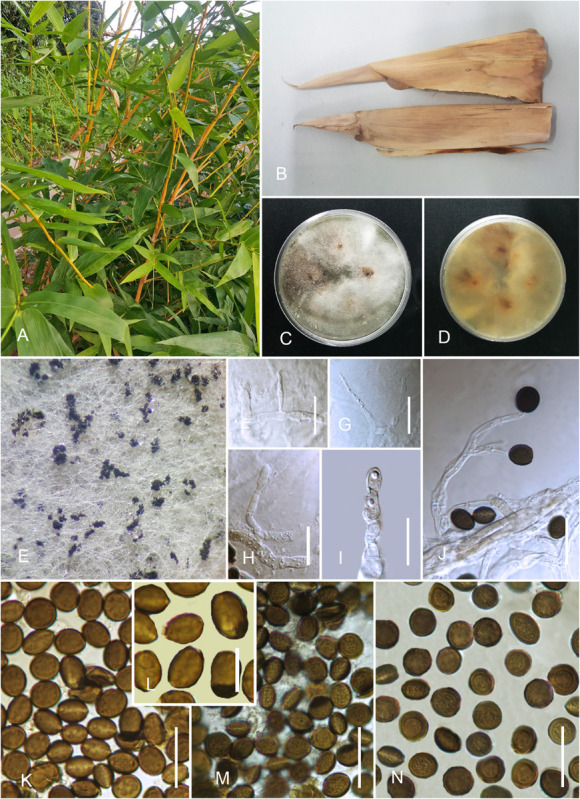
*Arthrinium pseudorasikravindrae* (HKAS 107669). **(A)** Host. **(B)** Fungarium specimen. **(C)** Surface view of culture on potato dextrose agar (PDA). **(D)** Reverse view of culture on PDA. **(E)** Conidial mass on cultures. **(F–J)** Conidia, conidiogenous cells, and conidiophores. **(K–N)** Conidia (concentric pale rings are arrowed in **I**). Scale bars: **(F–N)** = 10 μm.

Index Fungorum number: IF 557870
Etymology: Species epithet the morphological similarity of this collection to *Arthrinium rasikravindrae.*
Holotype: HKAS 107669

*Saprobic* on sheaths of *B. dolichoclada* Hayata. *Mycelium* 1.5–3 μm in diam., consisting of smooth, hyaline, septate, branched, hyphae. *Sexual morph*: undetermined. Asexual morph: *Conidiophores* 10–15 × 3–7 μm ($\bar{x}$ = 12.3 × 5.2 μm, *n* = 30), basauxic, straight or flexuous, cylindrical, hyaline, thick, smooth-walled, aseptate. *Conidiogenous cells* 4–10 × 1.2–5 μm ($\bar{x}$ = 8.6 × 4.2 μm, *n* = 30), holoblastic, develop from conidiophore mother cells, ampulliform, cylindrical or doliiform, hyaline to olivaceous. *Conidia* 5–10 × 5.5–11 μm ($\bar{x}$ = 9.3 × 10.1 μm, *n* = 30), globose in face view, lenticular in side view, with a pale longitudinal slit, dark brown, thick-walled, finely roughened with one or two concentric pale rings.

##### Culture Characteristics

Colonies grew on PDA at 20°C in the dark attenuated 2 cm diam., within 5 days, flat, spreading, circular, margin filiform with abundant aerial mycelia, surface white to off-white and reverse pale yellow, sporulation occurs on 2% PDA incubated at 25°C after 2 weeks, black, conidial mass concentrated at colony margins. Sporulation occurred after 10 days on PDA incubated at 20°C in the dark without any host substrate. Conidia seem black mass and spread mostly in colony margins.

##### Specimen Examined

China, Guangdong Province, Shenzhen City, Futian District, northwest of Futian, Bijiashan Park, on sheath of *B. dolichoclada* Hayata (Poaceae), 23 September 2018, IS, SI 73 (HKAS 107669, holotype), ex-type culture, KUMCC 20-0208; *ibid* October 15, 2018, IS, SI 73-1 (HKAS 107670, paratype), ex-paratype culture KUMCC 20-0211.

##### Notes

Blast results of ITS, LSU, β-tubulin, and tef 1-α sequences of *A. pseudorasikravindrae* (KUMCC 20-0208, KUMCC 20-0211) show high similarity to *A. hydei*, *A. paraphaeospermum*, and *A. rasikravindrae.* In our phylogenetic analysis, *A. pseudorasikravindrae* forms a subclade (subclade C1, Figure 1) with strong bootstrap support values (ML/PP = 90/0.96), which is a sister to the newly introduced species *A. acutiapicum.* Additionally, *A. pseudorasikravindrae* shows close phylogenetic affinities to *A. chinense*, *A. paraphaeospermum*, and *A. rasikravindrae* (Figure 1).

*Arthrinium pseudorasikravindrae* is morphologically distinct from the above species (Table 2) by its thick-walled, finely roughened conidia with pale, equatorial slit and ampulliform, cylindrical or doliiform, basauxic conidiogenous cells. The morphology of *A. pseudorasikravindrae* is compared with other closely related species (Table 2). Therefore, considering morphological and molecular uniqueness, these isolates are introduced here as belonging to a new species, *A. pseudorasikravindrae*. HKAS 107669 and HKAS 107670 represent a distinct clade (clade A, Figure 1) which not known before in the phylogenetic analysis, and hence, these collections are introduced here as a new species based on their morphology and phylogeny.

**TABLE 2:** Morphological comparisons of species which are phylogenetically closely related to *Arthrinium acutiapicum* and *Arthrinium pseudorasikravindrae*.

**Characters**	***A. acutiapicum***	***A. chinense***	***A. paraphaeospermum***	***A. pseudorasikravindrae***	***A. rasikravindrae***
Host/substrate	*Bambusa bambos/*dead twigs	*Fargesia qinlingensis*/culms	*Bambusa* sp./culms	*Bambusa dolichoclada*/sheath	Soil, *Coffea arabica*/leaf, *Pinus thunbergii*/wood, marine submerged wood, *Oryza granulate*/leaf
Localities	China	China	Thailand	China	Norway, China, Japan
Life mode	Saprobic	Saprobic	Saprobic	Saprobic	Saprobic, endophytic
Conidiophore	Reduced to conidiogenous cells	Reduced to conidiogenous cells	Reduced to conidiogenous cells	10–15 × 3–7 μm, cylindrical, branched, aseptate thick-walled hyaline	5-90 × 1-1.5 μm, arising from swollen basal cells, unbranched, septate, thin-walled hyaline to subhyaline
Conidiogenous cell	4–7 × 2–3 μm, cylindrical to ampulliform with pointed apex, pale brown, apex hyaline	1.5–6.5 × 1–3.5 μm, aggregated in clusters on hyphae, doliiform to clavate or lageniform, hyaline to pale brown	25–30 × 4–6 μm, aggregated in clusters on hyphae, elongated, conical to ampulliform, hyaline	4–10 × 1.2–5 μm, phialidic with periclinal thickening, ampulliform, cylindrical or doliiform, hyaline to olivaceous	5–10 × 2–4 μm, phialidic with periclinal thickening, ampulliform, hyaline
Conidia	Monomorphous, 7.5–10 × 8.5–12 μm, globose in surface view, subglobose to oval in side view, brown to dark brown, smooth, apex and base blunted, dark equatorial slit	Monomorphous, 8.5–11 × 6.5–8 μm, subglobose to lenticular, brown to dark brown, smooth to finely roughened, a longitudinal germ slit	Dimorphous, globose 10–19 × 11–20 μm, ellipsoid to clavate 20–30 × 9–13 μm, brown, smooth to somewhat granular, with pale equatorial slit	Monomorphous, 5–10 × 5.5–11 μm, globose in surface view, lenticular in side view, dark brown, thick-walled, with a pale equatorial slit, finely roughened, one or two concentric pale rings	Dimorphous, lenticular 10-15 × 6-10.5 μm, elongate to clavate 15-25 × 7.5-10 μm, arising acropleurogenously, brown to olivaceous, smooth, two-walled, prominent truncate base and equatorial germ slit
References	This study	Jiang et al. (2020)	Hyde et al. (2016)	This study	Singh et al. (2012)

## Discussion

Bamboo is an important group of flowering plants that helps to conserve and manage forest ecosystems and reduce soil erosion and it is also important for panda conservation and many more commercial applications such as making fishing rod, flute, paper, flooring material, etc. and as food for humans and livestock (Chapman and Peat, 1992). Members of bamboo belong to the family Poaceae comprising more than 115 genera with approximately 1,450 species (Gratani et al., 2008; Kelchner and Group, 2013), and bamboo occurs in all tropical, subtropical, and temperate regions as herbaceous or woody plants. Microfungi associate with bamboo in many ways and phytopathogenic or endophytic microfungi form diseases while saprobic microfungi help to decompose plant debris (Zhang and Wang, 1999; Hyde et al., 2002a,b).

The first monograph on bambusicolous fungi was published with 258 fungal species by Hino and Katumoto (1960), and 63 new species were introduced by Petrini et al. (1989). Eriksson and Yue (1998) provided a checklist of the ascomycetes on bamboo, while Zhang and Wang (1999) recorded 213 species described from bamboo in China. Kuai (1996) listed phytopathogenic bambusicolous fungi in China and Taiwan. Hyde et al. (2002a) reviewed bambusicolous fungi that grow on all bamboo substrates including the leaves, culms, branches, rhizomes, and roots and enlisted more than 1,100 species, which belong to 228 genera. Dai et al. (2018) have reviewed the taxonomy of bambusicolous fungi. This study is one of the articles in the series on bambusicolous microfungi in Guangdong Province. Herein, we collected *Arthrinium*-like taxa from bamboo plant samples from Shenzhen, Guangdong Province, China. Currently, there are 81 species in the *Arthrinium* (Species Fungorum, 2020) and only 61 have molecular data. More than 30% of holotypes of *Arthrinium* species have been collected in China (Table 1). Therefore, the aims of this paper were to study *Arthrinium*-like fungi in Guangdong Province and to introduce several putative new species by comparing them morphologically and genetically with existing taxa.

According to morphology and phylogeny, two novel *Arthrinium* species were obtained with two new locality records. Most phylogenetic studies on *Arthrinium* used ITS, β-tubulin, and tef 1-α; however, LSU has been added to the analyses here. Negligible variations occur in tree topology in spite of adding LSU. *A. guizhouense* (HKAS 107672) is the first record in Guangdong Province and also from bamboo. The holotype of *A. guizhouense* was collected from the air in kart caves in Guizhou Province, China (Wang et al., 2018). This suggests that fungal conidioma in plant hosts release the conidia and conidia can survive in the air for a sufficiently long time. Our strain of *A. bambusae* is identical to the holotype which was collected from Guangdong Province on bamboo (Wang et al., 2018). Hence, this specimen can be used as an epitype if the holotype cannot be used for taxonomic purpose. The morphological differences between these two *Arthrinium* species are listed in Table 2. However, the life mode, host, and colony characters of these two species are not significantly different.

## Data Availability Statement

The datasets generated in this study can be found in online repositories. The names of the repository/repositories and accession number(s) can be found in the article/supplementary material.

## Author Contributions

IS designed the study, performed the morphological study and phylogenetic analyses, and wrote the manuscript. JB, NX, and RC reviewed and edited the manuscript. All authors approved the final manuscript.

## Conflict of Interest

The authors declare that the research was conducted in the absence of any commercial or financial relationships that could be construed as a potential conflict of interest.

## Footnotes

1 http://www.indexfungorum.org

2 http://mafft.cbrc.jp/alignment/server/large.html

3 www.treebase.org
